# Effects of Vitamin E and Coenzyme Q_10_ Supplementation on Oxidative Stress Parameters in Untrained Leisure Horses Subjected to Acute Moderate Exercise

**DOI:** 10.3390/antiox10060908

**Published:** 2021-06-03

**Authors:** Alenka Nemec Svete, Tomaž Vovk, Mojca Bohar Topolovec, Peter Kruljc

**Affiliations:** 1 Small Animal Clinic, Veterinary Faculty, University of Ljubljana, Gerbičeva ulica 60, 1000 Ljubljana, Slovenia; alenka.nemecsvete@vf.uni-lj.si (A.N.S.); mojca.bohar@siol.net (M.B.T.); 2 The Chair of Biopharmaceutics and Pharmacokinetics, Faculty of Pharmacy, University of Ljubljana, Aškerčeva cesta 7, 1000 Ljubljana, Slovenia; tomaz.vovk@ffa.uni-lj.si; 3 Clinic for Reproduction and Large Animals, University of Ljubljana, Gerbičeva ulica 60, 1000 Ljubljana, Slovenia

**Keywords:** warm-blooded horses, acute moderate exercise, exercise-induced oxidative stress, coenzyme Q_10_, vitamin E, malondialdehyde, creatine kinase, aspartate aminotransferase, antioxidant enzymes

## Abstract

The effects of antioxidant supplements on exercise-induced oxidative stress have not been investigated in untrained leisure horses. We investigated the effects of 14-day supplementation with vitamin E (1.8 IU/kg/day), coenzyme Q_10_ (CoQ_10_; ubiquinone; 800 mg/day), and a combination of both (the same doses as in mono-supplementation) on the blood levels of CoQ_10_, vitamin E, and oxidative stress parameters in untrained leisure horses subjected to acute moderate exercise. Correlations between lipid peroxidation and muscle enzyme leakage were also determined. Forty client-owned horses were included in the study, with 10 horses in each of the antioxidant and placebo (paraffin oil) groups. Blood parameters were measured before supplementation, before and immediately after exercise, and after 24 h of rest. The differences in individual parameters between blood collection times and groups were analysed with linear mixed models (*p* ˂ 0.05). None of the supplemented antioxidants affected vitamin E and CoQ_10_ concentrations, oxidative stress parameters, or serum muscle enzymes. Lipid peroxidation occurred in horses supplemented with placebo and CoQ_10_ but not in horses supplemented with vitamin E or the combination of both antioxidants. These results suggest that vitamin E alone or in combination with CoQ_10_ prevented lipid peroxidation in untrained leisure horses subjected to acute moderate exercise.

## 1. Introduction

The beneficial effects of regular, non-exhaustive physical activity have long been known [1,2]. However, these beneficial effects are lost with strenuous, exhaustive, or unaccustomed exercise, which can lead to muscle damage, inflammation, and oxidative stress [3,4]. In humans and animals, exercise can lead to the increased production of reactive species, reactive nitrogen species (RNS), and especially reactive oxygen species (ROS), which can lead to oxidative stress and consequent oxidative damage to DNA, proteins and lipids, especially in untrained individuals [4,5,6,7,8,9]. On the other hand, physiological concentrations of ROS play an important role in cell signalling and in regulating gene expression and are thus essential for normal cellular functions [3,4]. In addition, moderate exposure to RNS and ROS is considered necessary for adaptation to exercise training by modulating muscle contraction and the activation of the endogenous antioxidant system, including the increased expression of antioxidant enzymes [4,8,9,10,11,12,13].

Organisms have evolved remarkably efficient antioxidant defence mechanisms to remove ROS and thereby prevent the damage caused by their action [6,7,14,15,16]. There is growing evidence that antioxidant defence systems, including enzymatic and non-enzymatic antioxidants, are capable of major adaptations during acute and chronic exercise [4,9,11,12,17,18]. Moderate exercise itself can be considered an antioxidant, as low to moderate concentrations of ROS act as signals that induce the expression of powerful endogenous antioxidant enzymes (superoxide dismutase (SOD), glutathione peroxidase (GPX), catalase, etc.) relevant to muscle cell adaptation to exercise as well as other defence mechanisms [3,4,11,18].

Physical activities of different intensities have been shown to induce oxidative stress not only in human athletes but also in horses; namely endurance horses [19,20,21,22,23], show jumpers and dressage horses [24,25], pentathlon horses [26], Marremmana racehorses [27], Thoroughbred racehorses [28,29,30], Standardbred trotters [31,32], and horses subjected to various moderate and intense treadmill exercise tests [33,34,35,36]. Lipid peroxidation is the most common consequence of exercise-induced oxidative stress and can lead to the release of muscle enzymes into the systemic circulation. Exercise-induced redox disturbances in skeletal muscle can also contribute to muscle fatigue and muscle injury [8,16]. In addition, lipid peroxidation induced by physical activity can also lead to myopathy and haemolysis [6,16,20,27,34,37,38,39].

Antioxidant supplements are commonly used to reduce exercise-induced oxidative stress, muscle damage, and inflammation and to enhance exercise performance in human athletes [3,4,7,8,11,13,40,41,42,43], as well as in sport horses [44,45,46,47,48,49]. However, the effects of antioxidant supplements are controversial in exercise research. Antioxidant supplementation can have both beneficial and pro-oxidant effects [4,7,44]. In addition, it may hinder or prevent important signalling adaptations to exercise training [12,13,50].

Vitamin E (α-tocopherol) is one of the most well-studied and widely used antioxidant supplements in exercise research in humans [8,13,41,50,51] and horses [34,45,47,49,52,53,54,55,56,57,58,59]. This exogenous antioxidant is one of the most potent free radical scavengers [60,61,62]. Vitamin E supplementation has been reported to reduce the concentration of lipid peroxidation products, indicating a protective effect of vitamin E supplementation against oxidative stress induced by various forms of physical activity in humans [63,64,65,66,67] and in sport horses [34,52]. On the other hand, a meta-analysis based on human studies showed that vitamin E supplementation did not result in significant protection against exercise-induced lipid peroxidation or muscle damage [41]. Similar results have been reported in sport horses [53,56,57,68]. The inconsistency of results regarding the effects of vitamin E supplementation on exercise-induced oxidative stress appears to reflect a variety of factors, including the amount, duration, form, and frequency of vitamin E supplementation; the type and timing of exercise; the age and fitness of the subjects; the vitamin E status of the subjects prior to the studies; and the methodology used to assess oxidative stress [8,41,50,58,66,69]. Nevertheless, it should be emphasised that there is limited evidence to support the use of vitamin E in human athletes [70].

Coenzyme Q_10_ (CoQ_10_; 2,3-dimethoxy-5-methyl-6-decaprenyl-1,4-benzoquinone), also known as ubiquinone, is the only endogenous fat-soluble antioxidant in the organism that is synthesised de novo. Coenzyme Q_10_ is known for its key role in mitochondrial bioenergetics. In addition, data showed that CoQ_10_ affects the expression of genes involved in human cell signalling, metabolism, and transport [71]. The reduced form of CoQ_10_, ubiquinol, is a potent antioxidant and may play a role in recycling tocopherols by effectively reducing the tocopheroxyl radical in mitochondria to alpha-tocopherol, thus regenerating the active form of vitamin E [71,72,73]. Considering the key role of CoQ_10_ in cellular energy production, CoQ_10_ has a potential role in the prevention of exercise-induced oxidative stress [43].

Several studies have reported beneficial, neutral, and adverse effects of CoQ_10_ supplementation on exercise-induced oxidative stress and exercise capacity [13,43,74,75,76]. These differences could be a consequence of inadequate CoQ_10_ doses and poor absorption after oral intake. Nevertheless, several studies investigating the effect of CoQ_10_ supplementation on exercise-induced oxidative stress in humans reported decreased levels of lipid peroxidation products [43,75,77,78,79]. Furthermore, ubiquinol supplementation minimised the exercise-induced depletion of CoQ_10_ and increased intracellular and plasma levels of antioxidants; however, it was unable to improve physical performance indices or reduce levels of creatine kinase (CK) and myoglobin, markers of muscle damage [76]. The discrepancy and inconsistency of results obtained in studies of CoQ_10_ supplementation may be due to differences between studies in CoQ_10_ formulations, dosages, the timing of supplementation, exercise tests performed, types of participants, and outcome measures used [13,43].

In contrast to human studies, there are few studies in the literature on CoQ_10_ supplementation in exercising racehorses [46,48,80]. However, there are no reports of CoQ_10_ as well as vitamin E supplementation in client-owned untrained leisure horses. Therefore, the aim of our study was to investigate the effects of 14 days of supplementation of vitamin E, CoQ_10_ (ubiquinone) and a combination of both on plasma concentrations of CoQ_10_ and vitamin E as well as on blood oxidative stress parameters (total antioxidant capacity (TAC), SOD, GPX, and malondialdehyde (MDA)) in untrained leisure horses subjected to acute moderate exercise. In addition, serum muscle enzymes, aspartate aminotransferase (AST) and CK, were determined to investigate possible correlations between lipid peroxidation product, MDA, and serum muscle enzymes.

## 2. Materials and Methods

### 2.1. Animals

Sixty-three client-owned, warm-blooded, untrained leisure horses were evaluated for inclusion in our randomised, double-blind, placebo-controlled study. Forty horses of both sexes (18 mares and 22 geldings) and different breeds, with a mean age of 10.6 years (range 3 to 18 years) and a mean weight of 544 kg (range 423 to 697 kg), met the inclusion criteria; young (less than 3 years) and old horses (more than 18 years), mares in foal, mares with foals, and competition horses were not included, as well as horses receiving antioxidant supplements and those with concurrent diseases. Horses were considered healthy based on history, results of physical examination, and results of haematological and biochemical analyses (Supplementary File). The horses were fed oats and hay. Water was provided ad libitum.

Written informed consent was obtained from the owners of the horses before inclusion in the study. All procedures were in compliance with the relevant Slovenian regulations (Animal Protection Act, Official Gazette of the Republic of Slovenia, No. 43/2007). The Ethical Committee of the Ministry of Agriculture, Forestry and Food, Veterinary Administration of the Republic of Slovenia approved all procedures (Licence No. 34401-5/2010/13).

### 2.2. Testing Protocol

Horses were randomly divided into 4 groups of 10 horses each and received supplements 2 times daily (at 7 a.m. and 19 p.m.) for 14 consecutive days. Horses in the CoQ_10_ group (G-CoQ_10_) received 800 mg/day of CoQ_10_ (ubiquinone; Q_10_ Vital Paste 7.5%, Valens Int. d. o. o., Šenčur, Slovenia) in the form of an inclusion complex with β-cyclodextrin (water soluble) produced in accordance with a previously filed patent [81]. Horses in the vitamin E group (G-Vit. E) received natural vitamin E (d-α-tocopherol acetate; natural vitamin E-oil, Natural Wealth, Bohemia, NY, USA) in the dose of 1.8 IU/kg BW/day [82], and the horses in the CoQ_10_ + Vit. E group (G-CoQ_10_ + Vit. E) received 800 mg CoQ_10_/day and vitamin E in the same dose as horses in the G-Vit. E group. The dose of vitamin E was calculated based on body weight; adult horses with an average body weight of 550 kg require 990 IU of vitamin E daily during moderate physical activity [82]. Horses in the control group (G-placebo) received 2.6 mL/day of paraffin oil (paraffinum liquidum, Ph. Eur. 6.0, series: MH163/10, 1 L, density at 20 °C, 0.865; Pharmachem sp., Ljubljana, Slovenia) as placebo. The calculated dose of paraffin oil was equivalent to a dose of a paste of 800 mg CoQ_10_/day.

### 2.3. Acute Moderate Exercise

All 40 horses performed the acute moderate exercise [82] in the manege (diameter of approximately 18 m) after 14 days of placebo and antioxidant supplementation and were supervised by a licensed trainer. The program included “alternative lunging” in the following order: 10 min walk (warm-up), 5 min slow trot, 10 min fast trot, and 15 min gallop. This was followed by a 15–20 min of cooling down by walk.

Respiratory rate, body temperature, and heart rate (Supplementary File) were measured immediately before and immediately after the exercise (40 min after the beginning of the exercise). Environmental factors air temperature and relative humidity were measured before the exercise.

### 2.4. Sample Collection and Preparation

Blood samples for the determination of haematological (Supplementary File), biochemical (Supplementary File), and oxidative stress parameters (TAC, SOD, GPX, and MDA) and antioxidants (vitamin E and CoQ_10_) were collected from the jugular vein before the first dose of supplements (basal values—0), after 14 days of supplementation (before exercise—1), at the end of the gallop (immediately after exercise—2), and 24 h after (3) the exercise.

Blood samples for the determination of haematological parameters and MDA were collected into 3 mL tubes containing K_3_EDTA, for biochemical parameters into 5 mL serum separator tubes and for TAC, SOD, GPX, vitamin E, and CoQ_10_ into five 2 mL tubes containing lithium heparin (all tubes Vacuette, Greiner, Kremsmünster, Avstria). Haematological analysis was performed within 5 h after collection of samples. Samples in serum separator tubes were left to clot for 60 min at room temperature and then centrifuged (1300× *g* for 10 min) to separate the serum. All biochemical parameters were determined immediately after serum preparation. Blood samples for the determination of plasma TAC, vitamin E, CoQ_10_, and MDA were centrifuged at 1500× *g* for 15 min at 4 °C. Plasma was separated, immediately frozen, and stored at −80 °C until analysis. Blood samples for the determination of GPX in whole blood lysates and SOD in washed red blood cells were prepared, immediately frozen, and stored at −80 °C until analysis.

### 2.5. Sample Analysis

Haematological analyses were performed with an Advia 120 automated laser haematology analyser (Siemens, Munich, Germany), biochemical parameters (except electrolytes—sodium, potassium, and chloride) were determined with an RX-Daytona automated biochemical analyser (Randox, Crumlin, England), and concentrations of electrolytes were determined with an Ilyte electrolyte analyser (IL—Instrumentation Laboratory, Lexington, USA). Concentrations of TAC, SOD, and GPX were determined spectrophotometrically with an RX-Daytona automated biochemical analyser (Randox, Crumlin, England) using commercially available reagent kits (total antioxidant status (TAS), Ransod (SOD) and Ransel (GPX); all Randox, Crumlin, England). MDA concentrations were determined by high-performance liquid chromatography (HPLC) and the electrospray ionisation method on an Agilent 6460 triple analyser with a triple quadrupole MS/MS detector (Agilent Technologies, Santa Clara, CA, USA). Plasma samples were treated with a derivatisation method using dinitrophenylhydrazine according to the literature [83]. Total CoQ_10_ concentrations were determined by HPLC and the atmospheric pressure chemical ionisation method on a Finnigan-LCQ mass spectrometer (Finnigan MAT, San Jose, CA, USA) according to the procedure described previously [84]. Vitamin E concentrations were determined using the HPLC method and an Alliance HPLC System 2695 fluorescence detector (Waters, Milford, MA, USA) according to the procedure described previously [85,86].

### 2.6. Data Analysis

Data were analysed using software package R (Version 3.0.2 for Windows; R Foundation for Statistical Computing, Vienna, Austria). Differences in individual parameters between blood collection times and groups of horses were analysed with linear mixed models [87,88], where fixed factors (fixed effects) take into account the blood collection, group of horses, and their interaction, and each horse was a candidate for the random intersection (random intercept). Multiple comparisons took into account the revised level of features for Bonferroni, *p* < 0.002. R-library was used with nlme mixed models and phia for the post hoc analysis by correcting the Holm–Sidak method. The Pearson correlation coefficient analysis was performed to determine the correlation between MDA and serum muscle enzymes (AST, CK). A value of *p* < 0.05 was considered significant. Data are shown as the means ± standard deviations (SD).

## 3. Results

No significant changes in plasma vitamin E (Table 1) or CoQ_10_ concentrations (Table 1) were observed in either group of horses between the different sampling times. However, a significantly higher plasma vitamin E concentration was observed in the CoQ_10_ + Vit. E group than in the placebo group at all sampling times. Except at the basal sampling time, plasma CoQ_10_ concentrations were significantly higher in the CoQ10 + Vit. E group than in the placebo group at all other sampling times.

Before supplementation, MDA concentrations (Table 2) did not differ significantly between horse groups. MDA concentrations increased significantly after exercise compared with concentrations before exercise in the placebo group and in the group of horses supplemented with CoQ_10_ (Table 2). In the placebo group, MDA concentrations remained significantly elevated even after 24 h of rest. However, no significant difference in MDA concentration was found between the placebo group and each group of horses supplemented with antioxidants at any of the sampling time points. Furthermore, MDA concentration was significantly higher in the CoQ_10_ group compared to the CoQ_10_ + Vit. E group after exercise.

Before supplementation, the TAC concentrations (Table 3) and activity of SOD (Table 4) did not differ significantly between the horse groups. During the study, the TAC levels (Table 3) and activity of SOD (Table 4) did not change significantly between the different sampling times in either group of horses. Moreover, we did not find significant differences in TAC values and SOD activities between the horse groups at any of the sampling times. On the other hand, the activity of GPX (Table 4) showed significant differences between the horse groups before supplementation. Compared to the placebo group, GPX activities were significantly high in horses supplemented with vitamin E and in horses supplemented with a combination of vitamin E and CoQ_10_ at all sampling time points. In horses supplemented with the combination of vitamin E and CoQ_10_, GPX activities were significantly increased after 24 h of rest compared to pre- and post-exercise activities.

Before supplementation, the activities of CK and AST (Table 5) did not differ significantly between the horse groups. In addition, we did not find significant differences in the activity of these two enzymes (Table 5) between the horse groups at any of the other sampling times. During the study, the activity of CK did not change significantly in either group of horses at the different sampling times. Regarding AST activity, we found a significant difference between sampling times 2 and 3 in the group supplemented with CoQ_10_ (Table 5). In the other groups of horses, no significant differences in AST activity were found between the different sampling times.

The results of statistical analyses showed no significant correlations between MDA and any of the serum muscle enzymes (AST, CK) in either group of horses at any of the sampling time points.

## 4. Discussion

To the authors’ knowledge, the present study is the first to report the effects of 14 days of supplementation with vitamin E, CoQ_10_, and a combination of both antioxidants on the plasma levels of CoQ_10_ and vitamin E and on blood oxidative stress parameters in untrained leisure horses subjected to acute moderate exercise. Contrary to our expectations, the supplementation of horses with vitamin E or CoQ_10_ did not result in increased plasma concentrations of vitamin E or CoQ_10_, respectively. However, supplementation with a combination of both antioxidants significantly increased plasma CoQ_10_ but not vitamin E concentrations. Although vitamin E and CoQ_10_ are known for their antioxidant properties and the sparing effect of CoQ_10_ on vitamin E, neither the individually supplemented antioxidants nor the co-supplementation of both affected oxidative stress parameters at any of the sampling time points.

In our study, untrained leisure horses were supplemented with 1.8 IU/kg/day of vitamin E as recommended for moderate physical activity for adult horses [82]. The dose of vitamin E used in our study was lower compared to vitamin E supplementation studies in sport horses. The doses used in these studies vary widely. In Thoroughbred and Quarter horses, vitamin E doses were 80 IU/kg dry matter (DM) and 300 IU/kg DM, respectively [53]; in sport horses, about 9 IU/kg/day (3 g alpha-tocopherol/day) [56]; in Standardbred horses, 10–20 IU/kg/day [57]; in Thoroughbred horses, 6 IU/kg/day [68]; and in exercising horses (trotters and riding horses) 1.5, 4.5, and 7.5 IU/kg/day (1, 3, and 5 mg of vitamin E/kg/day) [53,56,59]. Despite supplementation with higher doses of vitamin E for a longer period of time than in our study, the serum or plasma concentrations of vitamin E did not increase [53,56,57,68], which is in agreement with our results [53,56,57,68]. Moreover, serum or plasma concentrations of vitamin E decreased significantly after the supplementation period in exercising sport horses [53,56]. On the other hand, Saastamoinen and Juusela [59] reported a significant increase in serum vitamin E concentration after the long-term supplementation of sport horses with 3 and 5 mg/kg/day of vitamin E, but not after supplementation with 1 mg/kg/day. These authors suggested that sport horses in training require a daily supplement of 3 or 5 mg vitamin E/kg/day to increase the serum vitamin E concentrations. The absorption of vitamin E requires the presence of fat [62,89,90]. Therefore, a lack of fat in the diet of horses can lead to the poor absorption of vitamin E [57,90]. Not only the presence of adequate amounts of fats in the diet but also the dietary matrix itself and the concentration of lipoproteins have an influence on the absorption of vitamin E [62,89,90]. In contrast to our results, some studies conducted in sport horses showed a significant increase in plasma or serum vitamin E concentrations after vitamin E supplementation [49,51,52,58,59]. Untrained leisure horses included in our study are a much more diverse group of horses (different breeds, differences in level and frequency of physical activity, and different diets and environmental factors) compared to groups of sport horses. Therefore, differences between studies could be due to differences in the horses included in these studies, as well as differences in diets, the duration of supplementation, doses, and forms of vitamin E. The form and source of vitamin E supplementation is very important in horses because it significantly affects not only plasma vitamin E concentrations [91] but also the level of oxidative stress parameters in blood samples [49]. Serum vitamin E concentration significantly increased after 14 days of supplementation with synthetic vitamin E and natural source of vitamin E (micellized form of vitamin E; RRR α-tocopherol) at the dose of 4000 IU/day, and the micellized form of vitamin E was superior to the synthetic form. On the other hand, no significant increase in serum vitamin E concentration was observed in the control group supplemented with a similar dose of vitamin E (1000 IU/day) as in our study [49]. Similarly, Pagan and colleagues [91] reported that supplementation with the micellized form of vitamin E in Thoroughbred horses was superior in increasing plasma vitamin E concentrations compared to synthetic vitamin E (dl-alpha-tocopheryl acetate) and a natural source of vitamin E (d-alpha-tocopheryl acetate) [91]. The latter was used in our study. In some exercise studies in humans, vitamin E supplementation increased plasma vitamin E concentrations [50,65,92,93], whereas in many other studies, plasma vitamin E concentrations were not measured [8,92].

Overall, the lack of a significant increase in plasma vitamin E concentration in our study could be due to the inappropriate form of this antioxidant supplement, inadequate dose and short duration of supplementation, individual differences in leisure horses, and poor absorption after oral intake.

Similar to vitamin E, CoQ_10_ supplementation did not significantly increase plasma CoQ_10_ concentrations in our leisure horses. In Thoroughbred racehorses, significantly increased serum CoQ_10_ concentrations were found at days 30 and 60 of CoQ_10_ supplementation with the same CoQ_10_ dose as that used in our study [80]. The difference between the studies could be due to a longer period of supplementation [80], the use of different CoQ_10_ formulations, and differences in the horses included in the studies. Increased plasma CoQ_10_ concentrations were obtained after the exercise and supplementation of Thoroughbred racehorses with much higher daily doses of CoQ_10_, 1.9 g and 3.4 g, respectively [46]. Interestingly, in Thoroughbred racehorses, Thueson and colleagues reported significantly higher CoQ_10_ concentrations in skeletal muscle after 10 days of supplementation with 1000 mg of ubiquinol [48]. In humans, CoQ_10_ supplementation leads to an increase in plasma CoQ_10_ concentrations, the magnitude of which depends on the dosage, duration, and also the type of formulation [94,95,96]. Most studies in human athletes have found increased serum or plasma CoQ_10_ concentrations following supplementation with ubiquinone or ubiquinol [43,75,76,77,78,79,97,98]. The lack of a significant increase in plasma CoQ_10_ concentration after supplementation with this antioxidant in our study could be due to the short duration of supplementation, inadequate dose of CoQ_10_, and poor absorption. Being a lipophilic substance, the absorption of CoQ_10_ follows the same process as that of lipids in the gastrointestinal tract. The absorption mechanism for CoQ_10_ appears to be similar to that of vitamin E. Similar to vitamin E, the absorption of CoQ_10_ is enhanced in the presence of lipids. Therefore, the absorption of supplemental CoQ_10_ may be increased when taken with fatty meals, which was not the case in the group of leisure horses studied. Furthermore, in humans, plasma CoQ_10_ concentrations are highly dependent on plasma lipoproteins [94,99]. We may assume that the lack of fat in horse meal, as well as the low concentrations of lipoproteins in horse blood [100], could also lead to poor absorption and thus the lack of a significant increase in plasma CoQ_10_ after supplementation. In contrast to humans, high-density lipoproteins are the major lipoprotein fraction in horses, followed by low-density lipoproteins; the smallest proportion of lipoproteins belongs to the very-low-density lipoproteins [100].

Interestingly, the supplementation of leisure horses with a combination of vitamin E and CoQ_10_ resulted in a significant increase in plasma CoQ_10_ but not vitamin E concentrations. Our results are in partial agreement with the results of a double-blind, placebo-controlled study in marathon runners [101], in which co-supplementation with these two antioxidants resulted in a significant increase in plasma concentrations of both antioxidants. Zhou and colleagues also reported increased CoQ_10_ concentrations pre- and post-exercise in men following supplementation of CoQ_10_ and the combination of CoQ_10_ and vitamin E; however, the plasma vitamin E concentrations were not measured in their study [102]. In human blood, 95% of CoQ_10_ is present in its reduced form, as ubiquinol, a potent lipophilic antioxidant [94,103]. The latter is capable of regenerating vitamin E from tocopheroxyl radicals in mitochondria, resulting in sparing of vitamin E and consumption of ubiquinol [104,105]. Therefore, supplementation with a combination of these two antioxidants would also be expected to result in a significant increase in plasma vitamin E concentration [106]. Vitamin E and CoQ_10_ are both lipophilic antioxidants with similar absorption and transport mechanisms, which could influence the concentration of both antioxidants in plasma. We hypothesise that increased plasma CoQ_10_ concentration observed in our horses after supplementation with CoQ_10_ + Vit. E could be due to the antioxidant properties of vitamin E or a synergistic effect of co-supplementation [107].

Numerous studies in humans and horses have investigated the effects of antioxidant supplementation on exercise-induced oxidative stress with equivocal results. This is most likely due to the wide variety of exercise and/or supplementation protocols, including the length of the supplementation period and form of antioxidants and exercise selected by the research group, as well as differences in the fitness of the athletes and sport horses included in the studies [8,13,41,42,43,44,50,74]. According to the results of the statistical comparison of oxidative stress parameters between the supplemented groups of leisure horses and the placebo group, antioxidant supplementation had no effect on oxidative stress parameters at any of the measurement time points. However, a significant increase in MDA concentration after the acute moderate exercise in the placebo and CoQ_10_ groups but not in vitamin E and CoQ10 + Vit. E groups suggests that vitamin E alone or in combination with CoQ_10_ prevented lipid peroxidation.

Malondialdehyde is a reliable and the most commonly used marker for the overall lipid peroxidation level and thus for the presence of oxidative stress [108,109]. It is the most commonly used marker of the level of lipid peroxidation in exercise and sport studies [110]. Several studies have confirmed the increased lipid peroxidation during different forms of physical activity in sport horses [19,21,23,24,27,31,32,34,111]. Similarly, in the present study, plasma MDA concentration increased significantly after physical activity in both the placebo and CoQ_10_ supplemented groups, while no significant increase was observed in the vitamin E and CoQ_10_ + Vit. E groups. In the placebo group, plasma MDA concentration remained significantly elevated even after 24 h of rest, indicating a higher extent of lipid peroxidation than in the CoQ_10_ group, although we found no significant difference in MDA concentration between the placebo and CoQ_10_ groups. These results may suggest that vitamin E and the combination of vitamin E and CoQ_10_ decreased the process of lipid peroxidation during exercise due to the antioxidant properties of vitamin E. Our findings are in agreement with the results of some studies that investigated the effects of vitamin E supplementation on exercise-induced oxidative stress in sport horses [51,57,68] and in humans [41,67]. The results of the meta-analysis showed that tocopherol supplementation did not result in significant protection against either exercise-induced lipid peroxidation or muscle damage. The lack of protective effects in general was explained by the complex behaviour of tocopherols, which can act as antioxidants, pro-oxidants, and neutral agents in vivo, and (or) possibly by insufficient accumulation in muscle tissue after supplementation [41]. Pro-oxidant properties have been demonstrated in highly trained human athletes, in which vitamin E supplementation resulted in an even greater increase in lipid peroxidation products than placebo supplementation after exhaustive exercise [93]. On the other hand, several studies in human athletes [63,64,65,67,92] and sport horses [34,52,58] have shown decreased levels of lipid peroxidation products following vitamin E supplementation, suggesting an antioxidant effect of vitamin E.

In contrast to our results, in most human studies, CoQ_10_ supplementation significantly reduced the concentration of lipid peroxidation products after different types of physical activities [43,75,76,77,78,79,112], indicating the antioxidant effect of CoQ_10_. In these studies, CoQ_10_ supplementation resulted in a significant increase in plasma CoQ_10_ concentration, which was not the case in our study.

The total antioxidant capacity is a biochemical parameter suitable for assessing the overall antioxidant status of serum or plasma resulting from the uptake and/or production of antioxidants and their consumption during normal or increased oxidative stress. The capacity of known and unknown antioxidants and their synergistic interaction are assessed, thus providing insight into the delicate balance between oxidants and antioxidants in vivo [113,114]. Supplementation with antioxidants can increase plasma TAC concentrations [114,115,116]; however, in our study, TAC concentrations remained unaffected by supplementation with any of the antioxidants at any of the sampling time points. Similar results were obtained by Duberstein and colleagues, who investigated the effect of training and vitamin E supplementation on oxidative stress parameters in exercising horses and used the same method for TAC determination [68], as well as in several exercise studies in human athletes supplemented with vitamin E [67], CoQ_10_ [77], and a combination of vitamin E and CoQ_10_ [101]. In contrast to our results, some human studies found increased TAC levels after supplementation with CoQ10 and strenuous exercise [75,79]. The lack of a significant increase in TAC levels after supplementation in our study could simply be due to the fact that there was no significant increase in plasma vitamin E and/or CoQ_10_ concentrations after supplementation or in the species used in our study. The latter could differ in the metabolism of the supplemented antioxidants compared to humans. In addition, the lack of a significant increase in plasma TAC concentrations could be partly due to methodological reasons. The methods used for TAC determination vary greatly, meaning that the results of different methods are not comparable [113,114,117].

In our study, TAC values did not show significant changes between different sampling times in any group of horses. Similarly, Balogh and colleagues found no significant differences in TAC levels before or after exercise in pentathlon horses, using the same method for TAC determination as in our study. However, in this study [26], TAC values measured by the method FRAP (ferric reducing ability of plasma) were significantly higher immediately after exercise than before exercise, supporting the idea of measuring TAC with two different methods rather than one. Our findings are similar to the results of studies conducted in sport horses [35,118], in which the chosen physical activity was not intense enough to cause the release of antioxidants that contribute to the increase in plasma or serum TAC concentrations [18]. On the other hand, Duberstein and colleagues reported significantly lower TAC levels after a standard exercise test in placebo and vitamin E supplemented horses [68]. Levels returned to baseline by 48 h after exercise. In addition, Duberstein and colleagues showed increased TAC concentrations in placebo and vitamin E-supplemented horses after five weeks of training, indicating that fitness must be considered when comparing TAC concentrations in exercising horses [68]. Significantly lower TAC levels were also reported in endurance horses at the midpoint (80 km) of the 160 km ride [19]. During and immediately after exercise, TAC may be temporarily lowered, as its components are used to quench the ROS produced. Later, during recovery, antioxidants are released from adipose tissue and the liver, and TAC concentrations usually rise above basal levels. As with other markers, studies reporting no change in antioxidant capacity after exercise, as was the case in our study, may have missed such changes because only one sample was taken immediately after exercise [7].

It is generally agreed that regular endurance training promotes the upregulation of antioxidant genes, leading to an increase in both total SOD and GPX activity in active skeletal muscle. Furthermore, high-intensity exercise training is superior to low-intensity exercise training in terms of the upregulation of muscle SOD and GPX activities [3,4,12,13,18]. Specific antioxidant enzyme activity has been shown to respond similarly to the TAC response to exercise. The antioxidant defence system may be temporarily reduced in response to increased ROS production but may increase again during the recovery period as a result of the initial pro-oxidant insult [7].

In horses, changes in the activity of the primary antioxidant enzymes, SOD and GPX, in response to exercise vary in the literature. In sport horses, significantly higher [19,20,29,57,119,120] or significantly lower activities [24,28,111] of blood antioxidant enzymes, either SOD and/or GPX, have been found after exercise as a result of increased ROS production, either by the upregulation of enzyme activity or by the increased utilisation of antioxidant enzymes to counteract ROS [3,9,13,18]. Moreover, no significant changes in antioxidant enzyme activities have been reported in sport horses [26,28,35,36,121,122]. Similarly, our study showed no significant changes in SOD and GPX activities post-exercise in comparison with pre-exercise values in any of the horse groups. It seems that in our study, the acute moderate exercise was not intense enough to induce changes in the activity of GPX and SOD. Conflicting results regarding changes in blood SOD and GPX activities could reflect differences in the intensity, duration, and type of exercise or training, as well as being due to differences in the timing of sampling and large individual differences between horses participating in the studies [6,7,44]. Similar conflicting results have been reported in exercise research in humans [7,69].

In our study, we found significantly higher GPX activity in the groups supplemented with vitamin E and with the combination of both antioxidants compared with the placebo group at all the measurement time points, which could be due to large interindividual differences in the activity of this antioxidant enzyme in our leisure horses. Nevertheless, we observed no significant effect of antioxidant supplementation on SOD and GPX activities. Similar results were found in sport horses after supplementation with CoQ_10_ (unpublished data) and vitamin E [28,52,57] and in humans after supplementation with CoQ_10_ [75,79].

Associations between exercise-induced oxidative stress and muscle enzymes leakage have been previously confirmed in sport horses [19,20,54,119]. Our study included untrained leisure horses, so we expected an increase in the activity of the serum muscle enzymes CK and AST, indicating some degree of muscle damage. However, we did not find a significant difference in the activities of AST and CK between pre- and post-exercise sampling times in any of the horse groups, suggesting that our leisure horses respond to acute moderate exercise without the presence of muscle damage that would lead to increased leakage of muscle enzymes into the circulation.

Furthermore, the activities of AST and CK were not affected by any of the antioxidant supplements. In addition, we did not find significant correlations between the lipid peroxidation marker MDA and serum muscle enzymes in any of the groups of leisure horses. A meta-analysis showed no significant effect of vitamin E supplementation on muscle damage in human athletes [8,41]. On the other hand, Helgheim and colleagues reported no significant increases in serum muscle enzyme activity in trained individuals after heavy exercise and a significant increase in CK in the untrained muscle group. Vitamin E supplementation had no effect on serum muscle enzymes after exercise in the trained muscle group [123]. Similar to our results, CoQ_10_ supplementation had no effect on CK activity in moderately trained healthy men [74].

## 5. Conclusions

Contrary to our expectations, supplementation with vitamin E and CoQ_10_ did not increase the plasma concentrations of these two antioxidants. However, supplementation with a combination of CoQ_10_ and vitamin E increased plasma CoQ_10_ concentrations compared with those of the placebo group. As indicated by a significant increase in MDA concentrations after acute moderate exercise, lipid peroxidation occurred in horses supplemented with placebo and CoQ_10_ but not in horses supplemented with vitamin E or the combination of vitamin E and CoQ_10_. These results suggest that vitamin E alone or in combination with CoQ_10_ prevented lipid peroxidation. However, we did not find a correlation between lipid peroxidation and serum muscle enzymes. Our results warrant further studies in larger groups of leisure horses using different dosing regimens and antioxidant formulations to determine the effect of antioxidant supplementation on exercise-induced oxidative stress.

## Figures and Tables

**Table 1:** Plasma vitamin E (µmol/L) and coenzyme Q_10_ (mg/L) concentrations (mean ± SD) in individual groups of horses during the study.

	Blood Sample Collection			
*Group*	0	1	2	3
G-placebo (n = 10) Vitamin E Coenzyme Q_10_	4.75 ± 1.52 2.94 ± 0.82	4.69 ± 1.39 2.48 ± 0.55	4.71 ± 1.93 2.31 ± 0.45	4.64 ± 1.81 2.16 ± 0.43
G-CoQ_10_ (*n* = 10) Vitamin E Coenzyme Q_10_	4.77 ± 1.79 2.70 ± 0.35	4.91 ± 1.97 2.88 ± 1.18	4.91 ± 1.89 3.10 ± 1.11	4.82 ± 1.93 2.51 ± 0.62
G-Vit. E (*n* = 10) Vitamin E Coenzyme Q_10_	3.63 ± 0.79 2.47 ± 1.20	3.87 ± 0.98 2.10 ± 0.50	3.66 ± 0.98 2.16 ± 0.43	3.59 ± 0.96 2.21 ± 0.60
G-CoQ_10_ + Vit. E (*n* = 10) Vitamin E Coenzyme Q_10_	7.73 ± 2.87 * 3.17 ± 0.88	8.43 ± 3.41 * 5.04 ± 1.92 *	8.19 ± 3.38 * 4.07 ± 2.36 *	8.18 ± 3.43 * 4.49 ± 0.69 *

* *p* < 0.05 compared to the placebo group. 0, before adding antioxidant supplements; 1, before acute moderate exercise; 2, immediately after acute moderate exercise; 3, 24 h after acute moderate exercise; G-placebo, control group of horses supplemented with placebo; G-CoQ_10_, group of horses supplemented with CoQ_10_; G-Vit. E, group of horses supplemented with vitamin E; G-CoQ_10_ + Vit. E, group of horses supplemented with combination of CoQ_10_ and vitamin E; *n*, number of horses included in the group.

**Table 2:** The MDA concentrations (µmol/L) (mean ± SD) in individual groups of horses during the study.

	Blood Sample Collection			
*Group*	0	1	2	3
G-placebo	4.99 ± 0.88	4.26 ± 0.95	6.29 ± 2.33 ^a^	6.34 ± 2.06 ^a^
G-CoQ_10_	5.66 ± 0.90	5.98 ± 1.15	7.55 ± 2.31 ^a,^**	6.33 ± 1.81
G-Vit. E	5.16 ± 0.60	5.71 ± 1.37	5.53 ± 1.68	5.34 ± 1.37
G-CoQ_10_ + Vit. E	4.94 ± 1.17	6.17 ± 2.05	5.13 ± 0.87	5.21 ± 1.67

^a^ *p* < 0.05 compared to blood collection time 1 (within group comparison); ** *p* < 0.05 compared to the G-CoQ_10_ + Vit. E group. 0, before adding antioxidant supplements; 1, before acute moderate exercise; 2, immediately after acute moderate exercise; 3, 24 h after acute moderate exercise; G-placebo, control group of horses supplemented with placebo; G-CoQ_10_, group of horses supplemented with CoQ_10_; G-Vit. E, group of horses supplemented with vitamin E; G-CoQ_10_ + Vit. E, group of horses supplemented with combination of CoQ_10_ and vitamin E; MDA, malondialdehyde.

**Table 3:** The TAC concentrations (mmol/L) (mean ± SD) in individual groups of horses during the study.

	Blood Sample Collection			
*Group*	0	1	2	3
G-placebo	1.22 ± 0.09	1.22 ± 0.07	1.26 ± 0.77	1.21 ± 0.05
G-CoQ_10_	1.22 ± 0.08	1.27 ± 0.12	1.28 ± 0.09	1.22 ± 0.09
G-Vit. E	1.14 ± 0.08	1.17 ± 0.08	1.18 ± 0.11	1.11 ± 0.08 ^b^
G-CoQ_10_ + Vit. E	1.16 ± 0.09	1.21 ± 0.06	1.23 ± 0.05	1.19 ± 0.08

^b^*p* < 0.05 compared to blood collection time 2 (within group comparison). 0, before adding antioxidant supplements; 1, before acute moderate exercise; 2, immediately after acute moderate exercise; 3, 24 h after acute moderate exercise; G-placebo, control group of horses supplemented with placebo; G-CoQ_10_, group of horses supplemented with CoQ_10_; G-Vit. E, group of horses supplemented with vitamin E; G-CoQ_10_ + Vit. E, group of horses supplemented with combination of CoQ_10_ and vitamin E; TAC, total antioxidant capacity.

**Table 4:** The SOD (U/g Hb) and GPX activities (U/g Hb) (mean ± SD) in individual groups of horses during the study.

	Blood Sample Collection			
Group	0	1	2	3
G-placebo SOD GPX	1809.8 ± 415.4 75.2 ± 30.2	1814.5 ± 263.7 76.0 ± 29.5	1707.1 ± 336.3 81.8 ± 33.1	1710.9 ± 384.2 83.7 ± 34.5
G-CoQ_10_ SOD GPX	1870.4 ± 360.9 56.0 ± 23.6 **	1894.5 ± 287.7 60.7 ± 24.4 **	1882.3 ± 328.8 63.1 ± 26.4 **	1915.7 ± 378.4 62.2 ± 25.3 **
G-Vit. E SOD GPX	1932.6 ± 234.9 204.6 ± 19.7 *^,^**	1915.6 ± 175.9 207.6 ± 21.6 *^,^**	1869.7 ± 200.2 212.2 ± 24.0 *^,^**	1897.0 ± 169.1 214.2 ± 20.2 *
G-CoQ_10_ + Vit. E SOD GPX	1898.0 ± 291.4 126.4 ± 44.1 *	1906.7 ± 290.6 126.8 ± 42.8 *	1999.8 ± 362.4 126.3 ± 41.6 *	1932.4 ± 337.1 198.3 ± 17.4 *^,a,b^

^a^ *p* < 0.05 compared to blood collection time 1 (within group comparison); ^b^ *p* < 0.05 compared to blood collection time 2 (within group comparison); * *p* < 0.05 compared to the placebo group; ** *p* < 0.05 compared to the CoQ_10_ + Vit. E group. 0, before adding antioxidant supplements; 1, before acute moderate exercise; 2, immediately after acute moderate exercise; 3, 24 h after acute moderate exercise; G-placebo, control group of horses supplemented with placebo; G-CoQ_10_, group of horses supplemented with CoQ_10_; G-Vit. E, group of horses supplemented with vitamin E; G-CoQ_10_ + Vit. E, group of horses supplemented with combination of CoQ_10_ and vitamin E; SOD, superoxide dismutase; GPX, glutathione peroxidase.

**Table 5:** The CK (U/L) and AST (U/L) activities (mean ± SD) in individual groups of horses during the study.

	Blood Sample Collection			
Group	0	1	2	3
G-placebo CK AST	181.0 ± 34.9 297.3 ± 50.5	180.3 ± 26.3 296.1 ± 47.0	218.2 ± 34.1 314.3 ± 43.8	204.7 ± 35.3 295.2 ± 40.8
G-CoQ_10_ CK AST	295.6 ± 113.5 350.1 ± 76.7	284.5 ± 110.3 356.6 ± 88.7	304.2 ± 115.0 372.2 ± 89.1	310.7 ± 126.6 346.1 ± 76.8 ^b^
G-Vit. E CK AST	225.8 ± 100.2 328.3 ± 87.6	242.9 ± 77.5 327.4 ± 76.1	267.7 ± 90.4 331.6 ± 74.6	256.4 ± 91.4 326.3 ± 71.3
G-CoQ_10_ + Vit. E CK AST	184.6 ± 51.6 293.8 ± 65.1	271.7 ± 121.3 309.8 ± 80.1	391.2 ± 175.3 328.2 ± 89.9	304.3 ± 167.4 317.5 ± 125.3

^b^ *p* < 0.05 compared to blood collection time 2 (within group comparison). 0, before adding antioxidant supplements; 1, before acute moderate exercise; 2, immediately after acute moderate exercise; 3, 24 h after acute moderate exercise; G-placebo, control group of horses supplemented with placebo; G-CoQ_10_, group of horses supplemented with CoQ_10_; G-Vit. E, group of horses supplemented with vitamin E; G-CoQ_10_ + Vit. E, group of horses supplemented with combination of CoQ_10_ and vitamin E; CK, creatine kinase; AST, aspartate aminotransferase.

## Data Availability

The data from this work are included in this article and in the Supplementary Materials and may be obtained from the corresponding author on reasonable request.

## Supplementary Materials

The following are available online at https://www.mdpi.com/article/10.3390/antiox10060908/s1. Supplementary file containing all results presented in the manuscript as well as results of haematological and biochemical analyses, body temperature, respiration, and heart rate in individual horses at different sampling time points.
